# HSP70 and their co-chaperones in the human malaria parasite *P. falciparum* and their potential as drug targets

**DOI:** 10.3389/fmolb.2022.968248

**Published:** 2022-08-05

**Authors:** Julian Barth, Tim Schach, Jude M. Przyborski

**Affiliations:** Biochemistry and Molecular Biology, Justus-Liebig University Giessen, Giessen, Germany

**Keywords:** chaperones, malaria, Plasmodium falciparum, Hsp40, Hsp70, protein-protein interaction, small molecule inhibitors, heat shock proteins

## Abstract

As part of their life-cycle, malaria parasites undergo rapid cell multiplication and division, with one parasite giving rise to over 20 new parasites within the course of 48 h. To support this, the parasite has an extremely high metabolic rate and level of protein biosynthesis. Underpinning these activities, the parasite encodes a number of chaperone/heat shock proteins, belonging to various families. Research over the past decade has revealed that these proteins are involved in a number of essential processes within the parasite, or within the infected host cell. Due to this, these proteins are now being viewed as potential targets for drug development, and we have begun to characterize their properties in more detail**.** In this article we summarize the current state of knowledge about one particular chaperone family, that of the HSP70, and highlight their importance, function, and potential co-chaperone interactions. This is then discussed with regard to the suitability of these proteins and interactions for drug development.

## Introduction

Malaria is one of the leading infectious diseases worldwide. The most lethal form is caused by *Plasmodium falciparum* (*P. falciparum*) which caused 241 million cases in 2020. The African continent accounted for up to 95% of these cases. Children under 5 years of age represent the most vulnerable group to the disease and account for 80% of the 627.000 deaths reported in 2020 (WHO, 2021).

Similar to other organisms, *Plasmodium* encodes a wide variety of HSP and other chaperones/co-chaperones which are involved in many essential cellular processes. These proteins play (or are predicted to play) a major role in the survival, virulence and pathogenicity of the parasite. As they lie at the heart of proteostasis, they assist in protecting parasite proteins in several situations of proteotoxic stress, including the temperature spikes caused by febrile episodes of the human host, temperature transitions taking place during transmission from the mosquito vector to the human host and *vice-versa*, and exposure to cytotoxic drugs. Due to their central role in such a diverse number of essential biological processes, these proteins have gained interest as potential targets for development of small molecule inhibitors. Several HSP have been shown to be upregulated in response to various drug treatments, and may play a role in helping parasites survive these stress situations (Akide-Ndunge et al., 2009; Cheeseman et al., 2012; Shahinas et al., 2013). Thus, as well as potentially being direct targets for drug development, any inhibitors identified may allow some measure of reversal of drug resistance.

Special interest has been paid to members of the HSP70 family, and their interactions with co-chaperones (HSP40, also known as J-domain proteins, JDP). The focus of this mini-review is to collate what is currently known about the biology of PfHSP70 and PfJDP, their interactions, and what progress has so far been made in developing specific inhibitors of this important parasite Achilles Heel.

## The HSP70 family

Chaperones of the HSP70-class are crucial elements of the cellular protein surveillance network. They are a highly conserved family of proteins that share a very similar structure. In general, they comprise an N-terminal nucleotide-binding domain (NBD) that is able to bind ATP. Following this is a protease-sensitive linker domain leading to a substrate-binding domain to which the corresponding substrate polypeptides bind (Flaherty et al., 1990). HSP70 are involved in diverse cellular processes such as protection from thermal insult, folding of nascent proteins, refolding of misfolded proteins, targeting terminally misfolded proteins for degradation and protein translocation.

## J-domain proteins

J-domain proteins (JDP, also referred to as HSP40, DNAJ) are generally co-chaperones for HSP70. They perform several tasks including recruitment of substrates to HSP70 and then stimulating the ATPase activity of HSP70. They can thus be viewed as adapters which allow a limited number of HSP70 to work on highly diverse substrates, and JDP are one of the most diverse co-chaperone families. In agreement with this, most organisms encode a higher number of JDP than HSP70 (Kampinga et al., 2019).

## The ATPase cycle and JDP-HSP70 interaction

**FIGURE 1:**
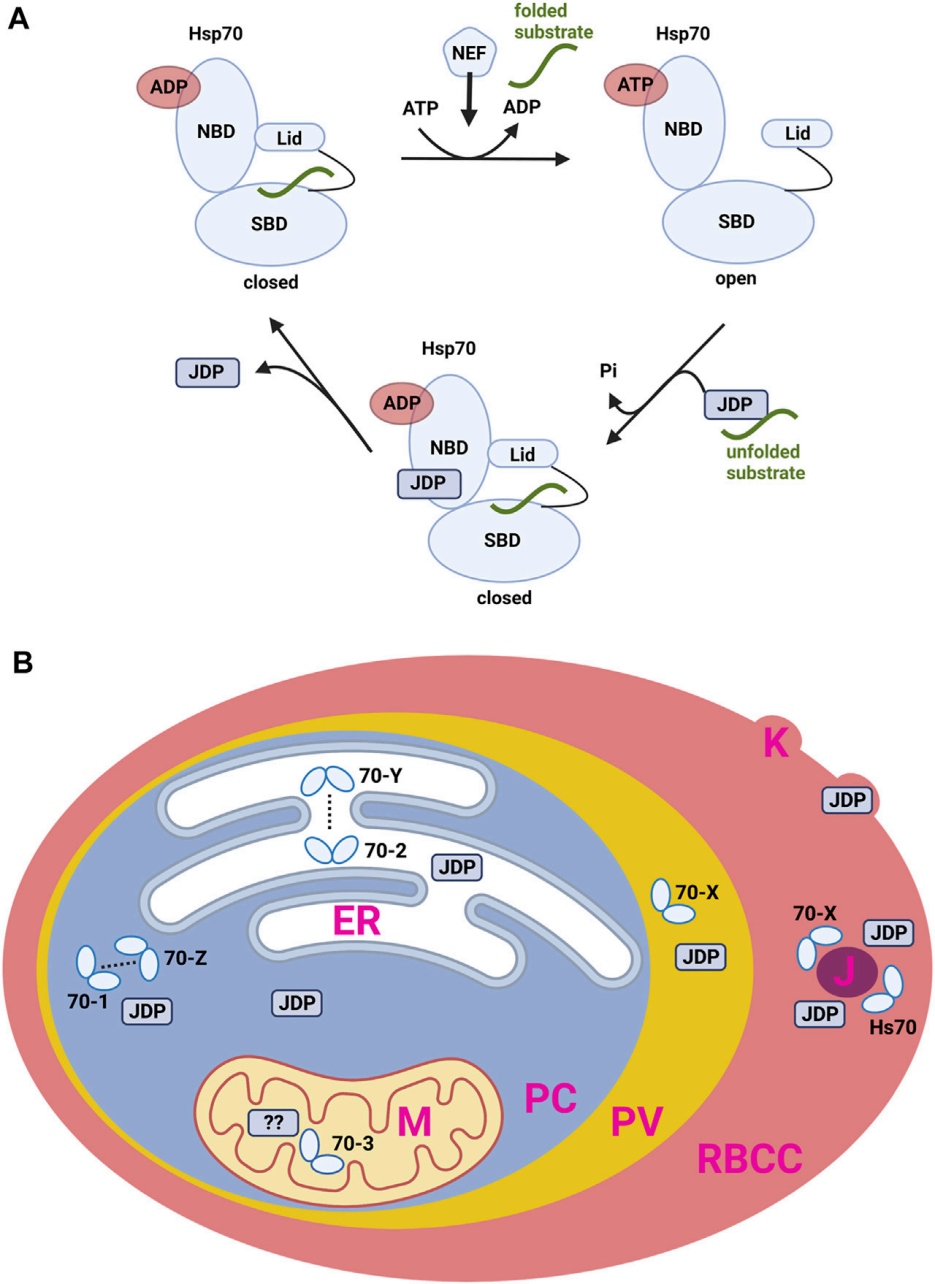
**(A)** The general ATPase cycle of HSP70. ADP, Adenosine di-phosphate; ATP, Adenosine tri-phosphate; NBD, nucleotide bindnig domain; SBP, subtrate binding domain; JDP, J-domain protein; Pi, inorganic phosphate; NEF, nucleotide exchange factor. **(B)** Localisation of HSP70 and JDP proteins in the P. falciparum-infected human erythrocyte. HSP70 are referred to by name as in text. PC, parasite cytosol; ER, endoplasmatic reticulum; M, mitochondrion; PV, parasitophorous vacuole; RBCC, red blood cell cytosol; J, J-dots; K, Knobs; JDP, J-domain protein.

HSP70 act as molecular chaperones. As such, they are able to bind and hold exposed hydrophobic peptide-sequences of other, aggregation-prone proteins. Beyond this “holdase” function, HSP70 are able to refold denatured proteins. A catalytic ATP-dependent interaction cycle enables the folding or refolding of substrate proteins. This essential cycle couples the ATPase activity of HSP70 to its affinity for substrate proteins (Figure 1A). In the ATP-bound state, the affinity to peptide substrates is low. In this context, a JDP binds first to a hydrophobic peptide-segment of any substrate protein and subsequently transfers it to HSP70 (Laufen et al., 1999; Mayer et al., 2000; Kityk et al., 2018). The simultaneous binding of a substrate to the SBD of HSP70 and a J-domain stimulate the ATPase activity of HSP70 synergistically (Figure 1A). Upon ATP-hydrolysis, the substrate-bound chaperone state is stabilized (Wittung-Stafshede et al., 2003). In the high-affinity HSP70-substrate-complex, the rate of ADP dissociation is the rate-determining step for the remainder of the cycle (Figure 1A). Nucleotide exchange factors (NEFs) facilitate ADP release and initiate ATP binding again with subsequent substrate release.

The enzymatic ability to hydrolyze ATP is essential for functional HSP70-substrate interaction (Mayer et al., 2000). Based on this mechanism, HSP70 are able to bind and protect virtually every protein from further denaturation and aggregation (Boorstein et al., 1994).

## The *P. falciparum* HSP70 and JDP families

Based on their structure and localisation, several PfHSP70 can be assigned functions by comparison to homologues in other systems (Figure 1B). Some members of the family have been more extensively studied and we now have some insight into their specialised function. Limited reverse genetic work has been carried out, however it is likely that the HSP70 (and some JDP) involved in core processes within the parasite will be essential for parasite survival, whereas those involved in (for example) host cell modification are not required in *in vitro* cell culture but may be important in an infection situation (Przyborski et al., 2015).

*PfHSP70-1:* PfHSP70-1 is likely to be the only canonical cytosolic HSP70. It contains a C-terminal -EEVD motif which is used for interaction with PfHOP (Zininga et al., 2015b). Although no definitive experimental evidence exists, inhibitor studies suggest that PfHSP70-1 has essential functions in the blood stages. Biochemical characterisation of recombinant PfHSP70-1 reveals that the protein has a slightly higher ATPase rate than that of the human homologue, but has a dramatically lower affinity for ATP (Matambo et al., 2004). PfHSP70-1 appears to be upregulated upon thermal stress (Kumar et al., 1991). PfHSP70-1 has been shown to associate with its putative NEF PfHSP70-Z in a nucleotide dependent manner (Zininga et al., 2015a; Zininga et al., 2016).

*PfHSP70-2:* PfHSP70-2 is localised in the ER, contains an N-terminal ER-type signal sequence and a C-terminal -SDEL ER retrieval sequence. PfHSP70-2 is likely to be a homologue of BiP/GRP78. PfHSP70-2 appears to be upregulated upon thermal stress (Kumar et al., 1991).

*PfHSP70-3:* PfHSP70-3 is likely to be targeted to the mitochondrion by virtue of an N-terminal transit peptide, but otherwise little is known about this protein.

*PfHSP70-X:* PfHSP70-X is only encoded by Plasmodium parasites belonging to the laverarian subgenus. These parasites infected humans, chimpanzees, and gorillas. PfHSP70-X locates to the lumen of the parasitophous vacuole and is also exported to the host cell (Külzer et al., 2012). In the host cell, this protein is found in structures referred to as J-dots, which also contain a number of parasite encoded JDP (Külzer et al., 2010; Külzer et al., 2012). Although partially exported to the host cell, the protein lacks a PEXEL export motif, and its transport appears to be directed by an atypical export signal found following an N-terminal ER-type signal sequence (Rhiel et al., 2016). Although not essential for parasite growth under normal conditions in culture, knockout experiments suggest that PfHSP70 is involved in a number of host cell modification processes including cytoadherance, antigenic variation and regulating the stiffness of the infected host cell (Charnaud et al., 2017). Knockdown experiments hint that PfHSP70-X may be involved in protecting the parasite from heat stress during fever periods (Cobb et al., 2017). Immunoprecipitation allowed the identification of a number of proteins interacting with PfHSP70-X, including both exported parasite proteins, a PV resident chaperone PfHSP101, human HSP70 and an exported parasite JDP (Zhang et al., 2017). The significance of this result is so far not known.

*PfHSP70-Y:* Also now known as PfGRP170, PfHSP70-Y belongs to the HSP110 protein family. These proteins are generally known to be NEFs for other members of the HSP70 family. PfGRP170 contains an N-terminal ER-type signal sequence and a C-terminal -KDEL ER retrieval sequence, and localises to the lumen of the ER. Earlier studies suggested that this protein may localise to the parasite’s apicoplast due to a predicted apicoplast transit peptide, however later work determined that the C-terminal -KDEL signal was dominant and retained the protein in the ER (Heiny et al., 2012). It is likely that PfHSP70-Y acts as a NEF for PfHSP70-2. The protein appears to be essential for parasite development and is linked to parasite stress responses (Kudyba et al., 2019).

*PfHSP70-Z:* PfHSP70-Z, also known as PfHSP110, belongs to the HSP110 protein family, and is likely to be the NEF for the cytosolic PfHSP70-1. Indeed, PfHSP70-Z has been shown to associate with PfHSP70-1 in a nucleotide dependent manner (Zininga et al., 2016). Recombinant PfHSP70-Z forms higher order oligomers and has been reported to have endogenous ATPase activity (Zininga et al., 2015a; Zininga et al., 2016). Functional inactivation of PfHSP70-Z is lethal, likely due to its role in preventing aggregation of a number of asparagine-rich proteins, especially under heat stress condition (Muralidharan et al., 2012). In agreement with this, expression of PfHSP70-Z is increased in response to heat stress (Zininga et al., 2015a).

*P. falciparum JDP*: *P. falciparum* encodes 43 proteins which can be assigned to the JDP family (Botha et al., 2007). Of these, 17 are predicted to be exported to the host cell, many of which are *P. falciparum* specific [not found in other non-laverania species (Botha et al., 2007)]. Based on the presence or absence of specific domains, the 43 JDP have been further assigned to a number of sub families, HSP40 Type I-Type IV. The 12 Type IV proteins are of particular interested as, although they contain a recognisable J-domain, the classical catalytic triad HPD has been replaced by HPE (Botha et al., 2007). This does not exclude a functional interaction with a HSP70, but implies that such interactions may be more specific and specialised. The function of the parasite-localised JDP has not been analysed in any great detail, but it is suggested that they likely act in concert with PfHSP70-1, PfHSP70-2 or PfHSP70-3. It is unknown why the parasite exports so many JDP. As JDP generally function in concert with a HSP70, it is supposed that these JDP functionally interact with either the exported PfHSP70-X, or potentially residual human HSP70/HSC70. A number of the exported JDP proteins have been knocked out, and many of these parasite lines show aberrations in host cell modification (Maier et al., 2008; Diehl et al., 2021). Of particular note, a knockout of the Type II exported JDP PFA66 shows dramatic changes in the morphology of the knobs (Diehl et al., 2021). Interestingly, further analysis suggested that this JDP functions in concert with residual human HSP70/HSC70. A model is emerging in which the parasite exports JDP to act as “adapter” molecules between parasite-encoded and residual host cell proteins (Diehl et al., 2021).

## The search for specific inhibitors of PfHSP70

A meaningful inhibitor would be specific for only Plasmodium PfHSP70, and its target and mode of action would be clear. A number of studies (detailed below) have reported inhibitors of PfHSP70 (Table 1). Some of these studies were carried out on recombinant protein, however the assays used are not always directly comparable, as they assay different sub-functions of HSP function. *In vitro* screening on parasite cell cultures has also been carried out, however it is not always clear what protein is being targeted. For inhibitors which have been assayed using both methods, there are often striking disparities between the effects on recombinant protein and in cell cultures. This suggests either off-target effects, or possibly limited bioavailability.

**TABLE 1:** Inhibitors so far tested against PfHSP70.

Substance		Biological effect			References
Classes	Compounds	*P. falciparum* IC_50_, effect on human cells	HSP70 effect	HSP70/JDP effect		
Pyrimidinones	MAL3-39	PfIC_50_ = 0.8 μM, HsIC_50_ = N/A	Weak inhibition of PfHSP70-1 and HSPA1A steady-state ATPase activity at 300 µM	Inhibitory effect on HSPA1A/Hdj2	Chiang et al., (2009) Botha et al., (2011)
	DMT-2264	PfIC_50_ = 1.1 μM, HsIC_50_ = N/A		Inhibitory effect on HSPA1A/Hdj2 and PfHSP70-1/PfHSP40	
Malonganenones	Malonganenone A	PfIC_50_ = 0.8 μM, Hs (MCF12A, MDA-231-MB, 50 µM) No effect	No inhibitory effect on basal ATPase activities of PfHSP70-X, PfHSP70-1 and HSPA1A	Strong inhibitory effect on PfHSP70-1/PfHSP40	Cockburn et al., (2011) Cockburn et al., (2014)
	Malonganenone B	PfIC_50_ > 50 μM, HsIC_50_ = N/A		All three compounds have a small significant inhi-bitory effect on PfHSP70-X/Hsj1a but no effect on HSPA1A/Hsj1a or PfHSP70-1/Hsj1a ATPase activity	Cockburn et al., (2011) Cockburn et al., (2014)
	Malonganenone C	PfIC_50_ = 5.2 μM, Hs (MCF12A, MDA-231-MB, 250 µM) No effect			
Naphtaquinones	Bromo-β-lapachona	PfIC_50_ = 17.3 μM, Hs (MCF12A, MDA-231-MB, 20 µM) 80% cell growth decrease	Strong basal PfHSP70-X ATPase activity inhibition, small inhibitory effect on HSPA1A, no effect on PfHSP70-1	Strong inhibitory effect on ATPase activity of PfHSP70-X/Hsj1a and PfHSP70-1/PfHSP40, no effect on HSPA1A/Hsj1a and PfHSP70-X/PFA066w_J_	Cockburn et al., (2011) Cockburn et al., (2014) Day et al., (2019)
	Lapachol	PfIC_50_ = 18.6 μM, Hs (MDA-231-MB, 200 µM) ∼ 50% cell growth decrease	Medium PfHSP70-X ATPase activity inhibition, no effect on PfHSP70-1 and HSPA1A	Medium inhibitory effect on PfHSP70-X/Hsj1a and PfHSP70-1/PfHSP40, no effect on HSPA1A/Hsj1a	Cockburn et al., (2011) Cockburn et al., (2014)
Chalcones	C86	PfIC_50_ = N/A, Hs (22Rv1, 5 µM) 55% cell viability decrease	No effect on basal PfHSP70-X ATPase activity. HsHSP70: N/A	Pre-incubation of PFE0055c with C86 results in inhibition of PfHSP70-X ATPase activity	Moses et al., (2018) Dutta et al., (2021)
(Benzothiazole)-Rhodacyanines	MKT-077	PfEC_50_ = 0.07 µM (3D7), HsEC_50_ = 0.98 µM (HCT-116)	Minimal PfHSP70-X ATPase activity inhibition under 100 µM HsHSP70: N/A	Small concentration-dependent inhibitory effect detected for PfHSP70-X/PFA066w_J_ and PfHSP70-X/PFE0055c_J_	Chen et al., (2018) Day et al., (2019) Dutta et al., (2021)
	YM-01	N/A		Concentration-dependent inhibitory effect on PfHSP70-X/PFA066w_J_ and PfHSP70-X/PFE0055c_J_	Day et al. (2019)
	JG98	PfIC_50_ N/A, HsIC_50_ ∼ 500 nM (22Rv1)	Significant PfHSP70-X ATPase activity inhibition at 10 µM HsHSP70: N/A	Significant inhibitory effect on PfHSP70-X/PFE0055c ATPase activity at 10 µM	Dutta et al. (2021)
Lipopeptides	Polymyxin B	PfIC_50_ = N/A, HsIC_50_ = varying, 1.05 mM (NRK-52E), 350 µM (HK-2)	Inhibition of basal ATPase and aggregation suppression activity of PfHPS70-1 and PfHSP70-z. HsHSP70: N/A	N/A	Azad et al. 2013) Zininga et al., (2017a)
Catechin	EGCG	PfIC_50_ = 2.9 μM, HsIC_50_ = varying, 22 µM (H661, H1299), 65 µM (HT-29)			Yang et al. 1998) Zininga et al., (2017b)
Bis-Indole	Violacein	PfEC_50_ = 400 nm (3D7), HsIC_50_ = 1.4 µM (HepG2)	Inhibition of basal ATPase and aggregation suppression activity of PfHSP70-1. HsHSP70: N/A		Bilsland et al*.* 2018 Tavella et al. 2021)

## Rational drug design

Modern drug discovery is moving more and more towards a rational design strategy based on knowledge of the target structure(s) and or interfaces. In infectious disease research, this often involves finding differences between proteins found in host and pathogen. Recent research findings focused on crystallographic elucidation of the functional domains of PfHSP70 and JDP (Day et al., 2019; Schmidt and Vakonakis, 2020; Mohamad et al., 2021). A study of the PfHSP70-X substrate-binding domain (SBD) reveals that the SBD-structure is conserved and extremely similar to both the human HSP70 (HsHSP70) and *E. coli* DnaK structure (Schmidt and Vakonakis, 2020). The NBD also shows a high conservation and similarity to that of the NBD of HsHSP70 (Mohamad et al., 2021).

While these results might lead to the conclusion that it is not viable to design inhibitors which specifically target PfHSP70-X while not affecting HsHSP70, the authors indicate that the NBD of PfHSP70-X does indeed contain potential binding sites for small-molecule activity-modulation which are structurally different in the human homologue. It is worth noting that crystallography can merely outline fixed protein structures whereas in the cell proteins (especially chaperones) are often conformationally dynamic. This flexibility influences their binding affinity to small molecules, allosteric modulators or other proteins (Johansson and Lindorff-Larsen, 2018). Thus, the use of structural information for the identification of specific small molecule inhibitors is complicated but a success is nonetheless possible.

## Characterised inhibitors of PHSP70

The search for small molecule inhibitors of the Plasmodium HSP70 chaperones has identified several suitable compounds. Amongst others, these compounds are pyrimidinones, malonganenones, naphtaquinones, lipopeptides, and a catechin from green tea extract (Chiang et al., 2009; Cockburn et al., 2011; Cockburn et al., 2014; Zininga et al., 2017a; Zininga et al., 2017b; Day et al., 2019). The activity-modulating effects towards several PfHSP70 chaperones are summarized in the following section.

### PfHSP70-1

As the main cytosolic chaperone of *P. falciparum*, PfHSP70-1 is heavily associated with maintaining viability and proteostasis and is thus a prominent target of molecular inhibitory research.

Members of the class of pyrimidinones exhibited varying effects on the intrinsic ATPase activity of PfHSP70-1 in single turnover assays. While they generally stimulated the ATPase activity at concentrations of 100 μM, the compounds DMT2264 and MAL3-39 inhibited the ATPase activity at higher concentrations of 300 µM (Chiang et al., 2009). First data regarding the malonganenones A-C, lapachol and bromo-*β*-lapachona (BBL) showed a concentration dependent inhibition of the aggregation suppression activity of PfHSP70-1 (Cockburn et al., 2011). However, the ATPase activity of PfHSP70-1 was not modulated by any of these compounds (Cockburn et al., 2014).

Studies with a focus on SPR analyses aim for the elucidation of binding affinities of potential small molecule inhibitors to several PfHSP70 chaperones. In this context, a screening of quinoline-pyrimidine hybrid molecules revealed varying binding affinities of these small molecules to PfHSP70-1 (Kayamba et al., 2021). Thus, the authors suppose that PfHSP70-1 is a target of these compounds as they exhibit moderate to high anti-plasmodial activity *in vitro*. Binding affinities for the green-tea polyphenol epigallocatechin-3-gallate (EGCG) and the lipopeptide polymyxin B (PMB) were defined in the same way (Zininga et al., 2017a; Zininga et al., 2017b). Additionally, both compounds inhibited the basal ATPase activity of PfHSP70-1 *in vitro*.

Furthermore, the phytocompounds iso-mukaadial acetate (IMA) and ursolic acid (UAA) feature anti-Plasmodium activity *in vitro* and *in vivo* (Nyaba et al., 2018; Salomane et al., 2021)*.* Both compounds were able to abrogate the aggregation suppression activity of PfHSP70-1 in a concentration dependent manner. The basal ATPase activity of PfHSP70-1 was inhibited by IMA similarly. UAA, however, did not modulate the ATPase activity in the highest tested concentrations significantly (Salomane et al., 2021).

Recently, the bis-indole violacein was tested for anti-malarial properties and possible inhibitory effects on PfHSP70-1. A significant and concentration dependent inhibition of the chaperone’s ATPase and aggregation suppression activity by violacein was observed (Tavella et al., 2021). Thus, the small molecule is predicted to compromise the ATP hydrolysis of PfHSP70-1 by interacting with the SBD or the SBD-NBD-interface (Tavella et al., 2021). However, as violacein exhibits broad biological activity, it also shows low selectivity for Plasmodium.

### PfHSP70-2

Data on small molecule inhibitors of PfHSP70-2 is limited. Four commercially available GRP78 inhibitors, namely Gilvocarcin V, Apoptozole, MKT-077 and VER-155008, have been reported to exhibit broad specificity to members of the HSP70-family (Matsumoto and Hanawalt, 2000; Rousaki et al., 2011; Kim et al., 2014; Park et al., 2017). They were confirmed to interact with PfHSP70-2 in *in vitro* binding assays, recently (Chen et al., 2018). However, the binding affinities of the compounds to PfGRP78 and HsGRP78 showed little difference across the species. An exception was VER-155008 that stood out due to a three-fold lower affinity to PfGRP78 than HsGRP78. The authors propose that the higher protein rigidity of PfGRP78 leads to lower affinities for this inhibitor. The marginally different properties of PfHSP70-2 possibly enable researchers to design compound derivates with specific inhibitory effects.

### PfHSP70-X

PfHSP70-X is of high interest in inhibitor research, as it is believed to assist the correct folding of exported proteins and thereby supporting parasite virulence. Similar to PfHSP70-1, the malonganenone-compounds did not modulate the basal ATPase activity of PfHSP70-X. However, napthaquinones, especially BBL, inhibited its ATPase and aggregation suppression activity in a concentration dependent manner (Cockburn et al., 2014). Regarding the ATPase activity, these findings were recently confirmed (Day et al., 2019). Additionally, the broad-spectrum HSP70 inhibitor MKT-077 attenuates the ATPase activity of PfHSP70-X only at concentrations above 100 µM. Its derivate YM-01 exhibits similar inhibitory properties at slightly lower concentrations (Day et al., 2019).

In contrary to the benzothiazole rhodacyanines JG98 and JG231, the chalcone C86 did not inhibit the basal PfHSP70-X ATPase activity significantly (Dutta et al., 2021). The authors evaluate their findings to be in accordance to the functional interaction of the compounds with PfHSP70-X. JG98 and JG231 are considered to prevent nucleotide exchange of an HSP70 and thus locking it in its ADP-bound form (Shao et al., 2018).

Comparing the varying results of studies with multiple small molecules and particularly PfHSP70-1 and PfHSP70-X, a striking distinction regarding their susceptibility to these compounds is observable. Thus, and according to Mohamad et al., 2021, it may be possible to design small molecules inhibitors to target specific PfHSP70s (Mohamad et al., 2021).

### PfHSP70-Z

Because PfHSP70-Z acts as a NEF for PfHSP70-1, inhibitors of the chaperones’ interaction have become a research target. The binding of the small molecules EGCG and PMB to PfHSP70-Z has been confirmed *via* SPR and *in vitro* activity assays showed an inhibitory effect of both compounds for the basal PfHSP70-Z ATPase activity. Additionally, EGCG and PMB interfere with the capability of PfHSP70-Z to suppress the aggregation of heat stress-prone proteins (Zininga et al., 2017a; Zininga et al., 2017b). Alternatively, a SPR screening of quinoline-pyrimidine hybrids suggested high binding affinities to PfHSP70-Z within the nanomolar and micromolar range for some compounds (Kayamba et al., 2021).

## Inhibitors of PfHSP70/PfJDP interaction

The explicated chaperone/co-chaperone interaction offers the possibility to inhibit a single part of the network and thereby achieving a loss of function in associated metabolic pathways (Daniyan et al., 2019). Especially the HSP70/JDP interface might be a viable target for controlling the PfHSP70’s ATPase activity *via* small molecule inhibitors (Day et al., 2019). The fact that small molecule compounds are able to modify the PfJDP-stimulated ATPase activity of their corresponding PfHSP70 is described in the literature for over a decade (Chiang et al., 2009; Botha et al., 2011; Cockburn et al., 2014). A number of compounds which have been shown to modulate intrinsic PfHSP70 activity have also been shown to modulate PfHSP70/PfJDP activities (Chiang et al., 2009; Cockburn et al., 2014; Day et al., 2019; Dutta et al., 2021). Similar strategies have been suggested in cancer research, in which HSP70/JDP activities have been associated with cancer cell progression (Nitika et al., 2020; Knighton et al., 2021).

First promising results were achieved by examining the human, Plasmodium and yeast HSP70 in combination with the human and yeast HSP40 co-chaperones Hlj1 and Ydj1, respectively (Chiang et al., 2009). Distinct and species-specific modulations of the HSP70 ATPase activity by a selection of nine pyrimidinones were reported. The capabilities of some particular compounds (MAL3-39 and DMT2264) were further assessed in a PfHSP70-1/PfHSP40 system (Botha et al., 2011). As a result, only DMT2264 was found to inhibit the PfHSP40 stimulated ATPase activity of PfHSP70-1.

Since the export of PfHSP70-X into the RBC has been shown, this particular chaperone gained further attention in small molecule compounds and JDP-interaction research (Külzer et al., 2012; Cockburn et al., 2014). Changes in PfHSP40-stimulated ATPase activity of PfHSP70-X and pfHSP70-1 in combination with lapachol, BBL and the malonganenones A (MA), B and C were monitored. The effects of the compounds were highly diverse. While BBL modulated the ATPase activities of PfHSP40/PfHSP70-1 and Hsj1a/PfHSP70-X, it also inhibited the ones of the Hsj1a/HSPA1A controls. Interestingly, MA provided selectivity of the HSP70’ modulation. The ATPase activity of the PfHSP40/PfHSP70-1 and Hsj1a/PfHSP70-X was significantly inhibited. The human controls, however, were not affected (Cockburn et al., 2014).

As more details on the interaction of PfHSP70 with specific PfHSP40 partners emerged, new experimental data on their inhibition was obtained (Daniyan et al., 2016; Day et al., 2019). Upon the simultaneous stimulation of PfHSP70-X by the J-domains of its supposed cognate co-chaperones PFA0660w_J_ or PFE0055c_J_, BBL did not decrease the ATPase activity of PfHSP70-X. Even broad-spectrum HSP70 inhibitors like methylene blue and MKT-077 showed little potency against PfHSP70-X that was stimulated by its *in vivo* co-chaperones (Day et al., 2019).

The chalcone C86 and the benzothiazole-rhodacyanines JG98 and JG231 were already identified as small molecule inhibitors of the human JDP/HSP70 system. Recently, they have been used on plasmodium proteins (Dutta et al., 2021). A significant inhibition of the stimulated ATPase activity of PfHSP70-X was observed when the JDP PFE055c was pre-incubated with C86 prior to its addition. This result fits the proposed function of C86 as a JDP pan-inhibitor (Moses et al., 2018). Additionally, JG98 and JG231 provided inhibitory effects on the PFE055c-stimulated ATPase activity of PfHSP70-X (Dutta et al., 2021).

These results imply that the development of parasite-specific chaperone/co-chaperone-based small molecule inhibitors is a complex task, but success can be achieved (Daniyan and Blatch, 2017).

## Conclusion

This mini-review summarizes the current findings of the search for Plasmodium HSP70 and HSP40 small molecule inhibitors. As these molecular chaperones are involved in multiple important molecular-biological processes of *P. falciparum*, they represent a promising new and sustainable drug target. Several studies successfully targeted and inhibited the PfHSP70’s and PfHSP40’s molecular chaperone activity in *in vitro* assays with small molecule inhibitors. However, the specificity of potential inhibitors is a critical point in research as human and Plasmodium chaperone counterparts share high structural similarity. Recent studies suggest that it may be possible to design specific small molecule inhibitors for PfHSP. Results of high-throughput screenings with derivates of already identified small molecule activity modulators and entirely new compounds are expected in the near future.
